# Atrial Fibrillation Beat Identification Using the Combination of Modified Frequency Slice Wavelet Transform and Convolutional Neural Networks

**DOI:** 10.1155/2018/2102918

**Published:** 2018-07-02

**Authors:** Xiaoyan Xu, Shoushui Wei, Caiyun Ma, Kan Luo, Li Zhang, Chengyu Liu

**Affiliations:** ^1^School of Control Science and Engineering, Shandong University, Jinan 250061, China; ^2^School of Information Science and Engineering, Fujian University of Technology, Fuzhou 350118, China; ^3^Department of Computing Science and Digital Technologies, Faculty of Engineering and Environment, University of Northumbria, Newcastle NE1 8ST, UK; ^4^The State Key Laboratory of Bioelectronics, Jiangsu Key Lab of Remote Measurement and Control, School of Instrument Science and Engineering, Southeast University, Nanjing 210096, China

## Abstract

Atrial fibrillation (AF) is a serious cardiovascular disease with the phenomenon of beating irregularly. It is the major cause of variety of heart diseases, such as myocardial infarction. Automatic AF beat detection is still a challenging task which needs further exploration. A new framework, which combines modified frequency slice wavelet transform (MFSWT) and convolutional neural networks (CNNs), was proposed for automatic AF beat identification. MFSWT was used to transform 1 s electrocardiogram (ECG) segments to time-frequency images, and then, the images were fed into a 12-layer CNN for feature extraction and AF/non-AF beat classification. The results on the MIT-BIH Atrial Fibrillation Database showed that a mean accuracy (Acc) of 81.07% from 5-fold cross validation is achieved for the test data. The corresponding sensitivity (Se), specificity (Sp), and the area under the ROC curve (AUC) results are 74.96%, 86.41%, and 0.88, respectively. When excluding an extremely poor signal quality ECG recording in the test data, a mean Acc of 84.85% is achieved, with the corresponding Se, Sp, and AUC values of 79.05%, 89.99%, and 0.92. This study indicates that it is possible to accurately identify AF or non-AF ECGs from a short-term signal episode.

## 1. Introduction

Atrial fibrillation (AF) is the most common type of arrhythmia in clinical disease and gradually becomes the world's rising healthcare burden [1]. According to Framingham heart study, lifetime risk of AF is about 25% [2]. The disease shows that the atrial activity is irregular, and the resulting complications such as stroke and myocardial infarction (MI) [3], endanger the health and lives of humans seriously [4]. Therefore, developing automatic AF detection algorithm is of great clinical and social significance [5, 6].

Generally, AF is significantly different from normal heart rhythm on electrocardiogram (ECG) signals [7]. During AF, RR interval is absolutely irregular and the P-wave is replaced by the continuous irregular F-wave, which is an important feature of AF [8]. Many scholars proposed diverse methods based on RR interval feature, but the accuracy of AF is not sufficient due to the complication of the ECG signals [9], and the pattern recognition ability of the existing statistical and general method is not satisfactory owing to a variety of noise interference [10].

In recent years, AF detection algorithms based on the time domain characteristics have been developed rapidly. Chen et al. [11] developed a multiscale wavelet entropy-based method for paroxysmal atrial fibrillation (PAF) recognition. In their work, recognition and prediction used support vector machine- (SVM-) based method. Fifty recordings from the MIT-BIH PAF Prediction Database were chosen to test the proposed algorithm, with an average sensitivity of 86.16% and average specificity of 89.68%. Maji et al. [12] used empirical mode decomposition (EMD) to extract P-wave mode components and corresponding parameters to determine the occurrence of AF. This proposed algorithm was tested with a total of 110 cycles of normal rhythm and 68 cycles of AF rhythm from the MIT-BIH AF Database. An average sensitivity of 92.89% and an average specificity of 90.48% were achieved. Ladavich and Ghoraani [13] constructed the Gaussian mixture model of the P-wave feature space. The model was then used to detect AF, with an average sensitivity of 88.87% and average specificity of 90.67%, while the positive predictive value was only 64.99%. Although relatively fine detection performances were achieved by the aforementioned methods, problems and questions exist. First, different methods used different signal length for AF identification. How will be the accuracy if performed on a very short-term (such as 1 s) ECG segment? This can show the ability for transient AF detection. In addition, ECG waveforms have various morphology and the abnormal waveforms are different when AF occurs, leading to poor generalization capability of the developed machine learning-based model. Thus, how to improve the model generalization capability is a key issue.

Convolutional neural networks (CNNs) [14] can extract features automatically without manual intervention and expert priori knowledge. Meanwhile, time-frequency (T-F) technology as a preprocess operation is to convert 1D ECG signals to 2D T-F features which can be used to transfer to a classifier. There are many common T-F methods at present, such as the short-time Fourier transform (STFT), the Wigner–Ville distribution (WVD), and the continuous wavelet transform (CWT) [15]. Luo et al. [16] presented a modified frequency slice wavelet transform (MFSWT) in 2017. MFSWT follows the rules of producing T-F representation and contains the information of ECG signals in both time and frequency domains, such as P-wave, QRS-wave, and T-wave. Additionally, MFSWT can locate the above characters accurately and avoid the complexity of setting parameters. The spectrogram of MFSWT can be expressed as images, while the combination of CNN and images is one of the most excellent choices. For example, Liu et al. [17] proposed the method to learn conditional random fields (CRFs) using structured SVM (SSVM) based on features learned from a pretrained deep CNN for image segmentation. Ravanbakhsh et al. [18] introduced a feature representation for videos that outperforms state-of-the-art methods on several datasets for action recognition. Lee and Kwon [19] built a fully connected CNN, which trained on relatively sparse training samples, and a newly introduced learning approach called residual learning for hyperspectral image (HSI) classification.

In this study, MFSWT was adopted to acquire T-F images for short-term AF and non-AF ECG segments from the MIT-BIH Atrial Fibrillation Database (MIT-BIH AFDB). A deep CNN with a total of 12 layers was developed to train an AF/non-AF classification model. Indices including accuracy, sensitivity, specificity, and the area under the curve were used for model evaluation based on a 5-fold cross validation method [20], to evaluate the stability and generalization ability of the proposed method in comparison with the existing methods. The existing research has achieved very good performance, but there is no validation for large data. In this paper, we used all the data in the database to increase the generalization ability of the model.

## 2. Methods

### 2.1. Modified Frequency Slice Wavelet Transform

In our previous work, modified frequency slice wavelet transform (MFSWT) [16] was proposed for heartbeat time-frequency spectrum generation, with following the major principle of frequency slice wavelet transform (FSWT) [21]. The modified transform generates T-F representation from the frequency domain, and a bound signal-adaptive frequency slice function (FSF) was introduced to serve as a dynamic frequency filter. The window size of FSF smoothly changes with energy frequency distribution of signal in low-frequency area. The MFSWT has good performance for low-frequency ECG signals, and its advantages include signal-adaptive, accurate time-frequency component locating. The reconstruction is also independent of FSF, and it is readily accepted by clinicians.

Assume $\hat{f}\left(k\right)$ is the Fourier transform of *f*(*t*). The MFSWT can be defined as follows:

(1) $$\begin{array}{c} W_{f}\left(t,\omega \right)=\frac{1}{2\pi }\int_{-\infty }^{+\infty }\hat{f}\left(k\right){\hat{p}}^{*}\left(\frac{\mu -\omega }{q\left(f\left(k\right)\right)}\right)e^{-ikt}dk, \end{array}$$

where *t* and *ω* are observed time and frequency, respectively, “*∗*” represents conjugation operator, and $\hat{p}$ is the frequency slice function (FSF):

(2) $$\begin{array}{c} \hat{p}\left(x\right)=e^{-x^{2}/2}. \end{array}$$

*q* is defined as a scale function of $\hat{f}\left(u\right)$,

(3) $$\begin{array}{c} q=\delta +\mathrm{sign}\left(\nabla \left|\hat{f}\left(\mu \right)\right.\right). \end{array}$$

It makes the transform to incorporate signal-adaptive property. In (3), *δ* corresponds to maximum $\left|\hat{f}\left(\mu \right)\right|$. ∇(·) is a differential operator, and sign(·) means signum function, which returns 1 if the input is greater than zero, 0 if it is zero, or −1 if it is less than zero. In (2), $\hat{p}\left(0\right)=1$, according to the claim in [21], then the original signal can be reconstructed as follows:

(4) $$\begin{array}{c} f\left(t\right)=\frac{1}{2\pi }\int_{-\infty }^{+\infty }\int_{-\infty }^{+\infty }W_{f}\left(t,\omega \right)e^{i\omega \left(t-\tau \right)}d\tau d\omega . \end{array}$$

**Figure 1:**
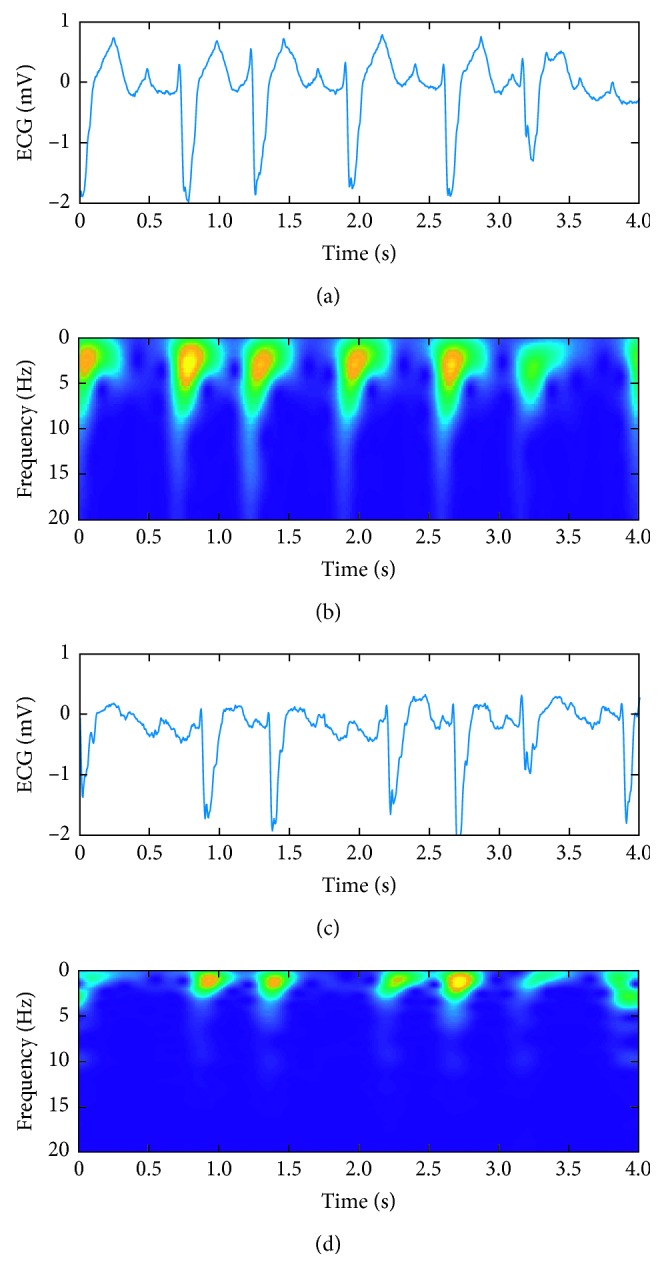
Examples from a 4 s normal ECG segment and a 4 s AF ECG segment, as well as their corresponding MFSWT spectra: (a) normal ECG signal; (b) MFSWT spectrum of the normal ECG signal; (c) AF ECG signal; (d) MFSWT spectrum of the AF ECG signal.

Figure 1 shows 4 s normal ECG, atrial fibrillation signals, and their corresponding MFSWT spectra, respectively, from 06426 recording. By the MFSWT, the time domain characteristics in ECG signal wave, such as P-wave, QRS-wave, and T-wave, have accurately been located in the signal spectrum. At the same time, each component of the spectrum of the T-F space distribution is corresponded well with the ECG signal frequency before.

In this study, the MFSWT is used as a tool to generate spectrograms of an ECG signal for CNN-based classification. The 1 s window, centered at the detected R-peaks (0.4 s before and 0.6 s after), was used to segment each heartbeat. Subsequently, the T-F spectrograms with the size of 250 × 90 (corresponded 1 s time interval and 0–90 Hz frequency range) were produced by the proposed MFSWT. This is then followed by data reducing. An average 5 × 2 template operator reduces the size of spectrograms to 50 × 45.

### 2.2. Convolutional Neural Networks (CNNs)

Deep CNN was improved by Lecun et al. [22]. CNN had breakthrough performance over the last few decades for solving pattern recognition problems [23], especially in image classification [24]. It has become a popular method for feature extraction and classification without requiring preprocessing and pretraining algorithm [25].

CNN is a composition of sequences of functions or layers that maps an input vector to an output vector. The input *x*_*k*_^*l*^ is expressed as follows:

(5) $$\begin{array}{c} x_{k}^{l}=\sum_{i=1}^{N_{l-1}}\text{conv}2\text{D}\left(w_{ik}^{l-1},s_{i}^{l-1}\right)+b_{k}^{l}. \end{array}$$

Similarly, *b*_*k*_^*l*^ and *w*_*ik*_^*l*−1^ are the bias and kernel of the *k*th neuron at layer *l*, respectively. *s*_*i*_^*l*−1^ is the output of the *i*th neuron at layer *l*−1, and conv2D(.  , .) means a regular 2D convolution without zero padding on the boundaries. So, the output *y*_*k*_^*l*^ can be described as follows:

(6) $$\begin{array}{c} y_{k}^{l}=f\left(\sum_{i=1}^{N_{l-1}}\text{conv}2\text{D}\left(w_{ik}^{l-1},s_{i}^{l-1}\right)+b_{k}^{l}\right). \end{array}$$

Besides, CNN also involves back-propagation (BP), in order to adjust the delta error of the *k*th neuron at layer *l*. Assuming that the corresponding output vector of the input is [*y*_1_^*L*^, *y*_2_^*L*^,…, *y*_*N*_*L*__^*L*^], and its ground truth class vector is [*t*_1_, *t*_2_,…, *t*_*N*_*L*__], we can write the mean absolute error (MSE) as follows:

(7) $$\begin{array}{c} E=E\left(y_{1}^{L},y_{2}^{L},\ldots ,y_{N_{L}}^{L}\right)=\sum_{i=1}^{N_{L}}{\left(y_{i}^{L}-t_{i}\right)}^{2}. \end{array}$$

Thus, the delta error can be concluded as

(8) $$\begin{array}{c} \Delta_{k}^{l}=\frac{\partial E}{\partial x_{k}^{l}}. \end{array}$$

**Figure 2:**
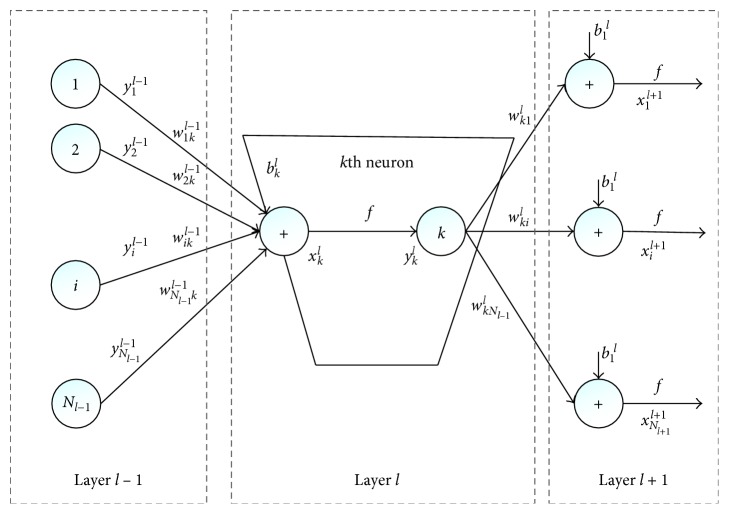
The implementation of CNNs.

The implementation of CNN [14] is as shown in Figure 2.

By the MFSWT, we have converted the signals to characteristic waves in a 2D space. Then, we use CNN to learn relevant information from the characteristic waves in a 2D space and achieve classification. The input to the CNN is characteristic waves in a 2D space computed from the exacted signals. The CNN was implemented using the Neural Network Toolbox in Matlab R2017a.

**Figure 3:**
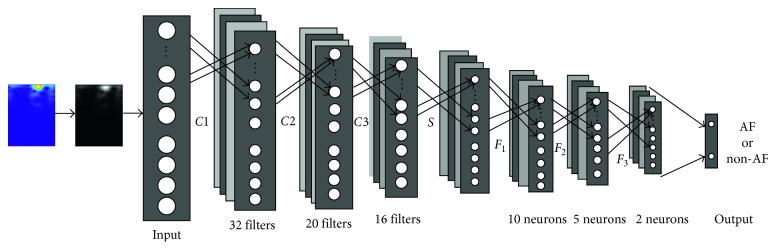
The architecture of the network.

In this paper, we use CNN to automatically extract the features of the labeled image and calculate the scores to classify the predicted image. A 12-layer network structure is developed, which contains 3 convolution layers, 3 ReLU layers, a max pooling layer, and 3 full-connection layers besides the input and output layers. We tested the effect of number of filters in each layer and obtained these values by running a grid search approach.  Figure 3 illustrates the architecture of the implemented network and its detailed components for each layer.

## 3. Experiment Design

### 3.1. Database

**Figure 4:**
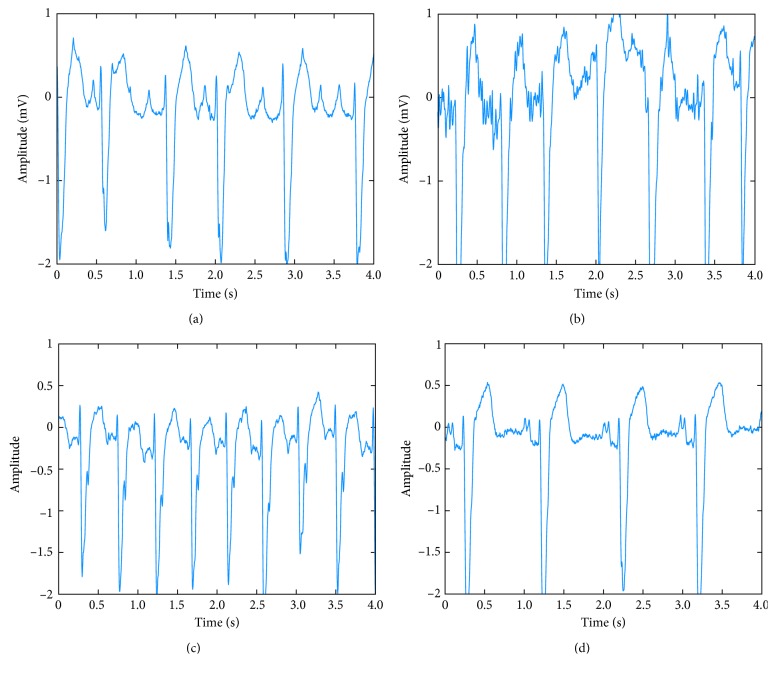
ECG examples of the four rhythm types: (a) normal, (b) atrial fibrillation, (c) atrial flutter, and (d) AV junction rhythm.

The database was from the MIT-BIH AFDB [26]. The MIT-BIH AFDB contains a large number of ECG data that have been annotated by a professional cardiologist, which is the authoritative ECG database in the classification of arrhythmia. This database may be useful for development and evaluation of atrial fibrillation/flutter detectors that rely on timing information only. It consists of 25 recordings (obtained from ambulatory ECG recordings of 25 subjects). The individual recordings are each 10 hours 15 min in duration and contain two ECG signals; each sampled at 250 samples per second with 12-bit resolution over a range of ±10 millivolts. The reference manual annotation files contain rhythm change annotations (with the suffix.atr) [27] and the rhythm annotations of types: AF, AFL (atrial flutter), J (AV junctional rhythm), and N (used to indicate all other rhythms) [28]. In our experiment, AFL, J, and N are attributed to non-AF category.  Figure 4 shows the ECG examples (each 4 s) of the four rhythm types (normal, AF, atrial flutter, and AV junction) from the “06426” recording.

### 3.2. Signal Preprocessing

There are a total of 25 recordings in AFDB, and two of them (numbers 00735 and 03665) have no relevant ECG data. Thus, 23 recordings are included in the experiment. In order to split the dataset equally, we divide the 23 recordings into five groups, and the basis for grouping is to reduce the differences of number in the two classes. The recording numbers for 5 groups are 5, 4, 5, 5, 4, subsequently. This experiment uses 5-fold cross validation for evaluation. For example, when using the first group to test, it means that the whole data in 04015, 04126, 04936, 07879, and 08405 recordings are used to verify, and the remaining 17 records are all used to train the model. Detailed recordings of grouping conditions are shown in Table 1.

We employ a balanced image dataset to train the model. That is, we choose the same number of AF samples from the non-AF category for training, while all the samples in the remaining fold for test. For example, for testing the fold 1, there are 415,109 normal images and 294,136 AF images as training data from the folds 2–5. We use all the 294,136 AF images and then randomly select 294,136 normal images, resulting in 588,272 images as training CNN model. Then, we test the performance of the developed CNN model using all data in fold 1, that is, 123,083 normal images and 90,403 AF images.  Table 2 presents the detailed numbers for each fold testing.

## 4. Results

### 4.1. Epoch Number of the CNN

**Figure 5:**
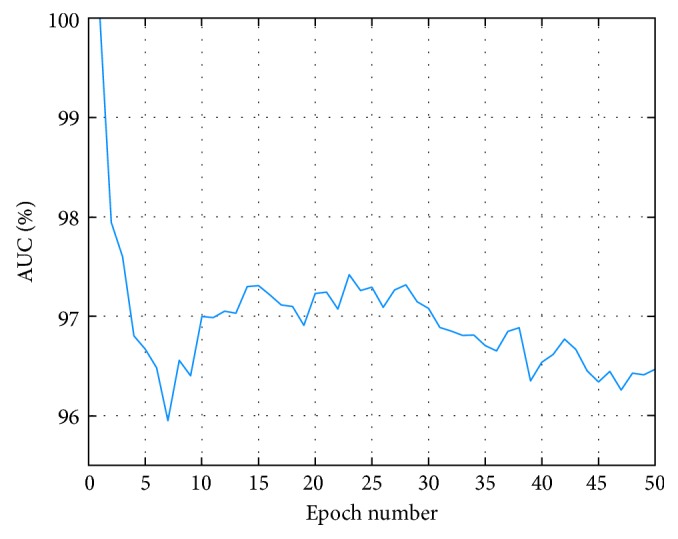
The AUC of test set at varying epoch number.

**Figure 6:**
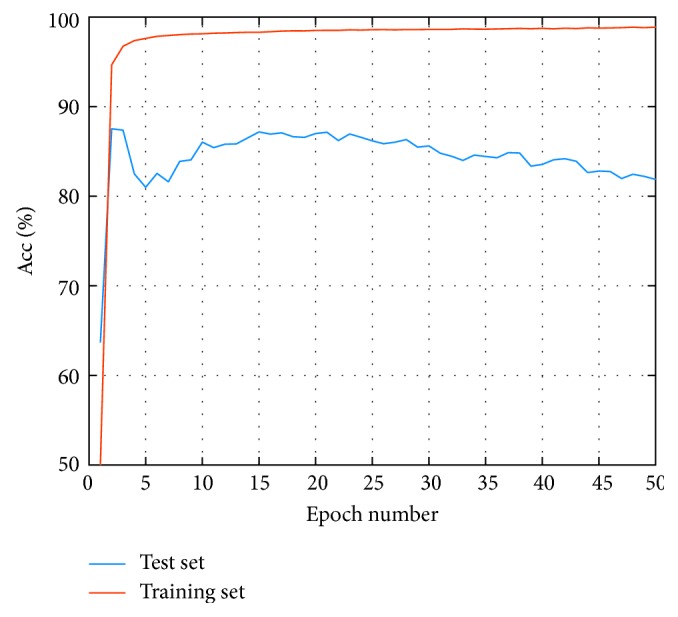
Test Acc and training Acc at varying epoch number.

The grid search method [29] is applied to select the optimal epoch number of the CNN.  Figure 5 shows the AUC of the test set (AUC is defined as the area under the ROC curve, often used to evaluate the classifier with imbalance data) at varying epoch number, and Figure 6 shows training and test accuracies at varying epoch number. We can see from Figure 5 that the AUC of test set is at a high level while epoch number is 15, and the wave becomes stable in Figure 6. Therefore, we choose the number of epochs as 15.

According to the introduction of Section 2.2 on CNN architecture, the input layer (layer 0) is for images with the size of 50 × 45 × 1 and is convolved with a kernel size of 10 × 9 to produce the layer 1. Layer 2 is the ReLU layer. The output of layer 2 is convolved with a kernel size of 8 × 7 to develop the layer 3 and layer 4. Similarly, a subsequent feature maps are convolved with a kernel size of 9 to acquire the layer 5 and layer 6. A max pool layer with the stride of 2 (layer 7) is applied to the generated characteristics. Then, the feature has been extracted. Finally, features are transported to layer 8 with 10 neurons and connected to 5 and 2 neurons in layer 9 and layer 10, respectively, to classify. In addition, we select the epoch number as 15, and the learning rate initial value is set to 0.001 while the number of minimal batches is 256. In addition, specific parameter is shown in Table 3.

### 4.2. Performance Metrics

This research uses the MIT-BIH AFDB to verify the proposed method to detect AF performance. Four widely used metrics, that is, sensitivity (Se), specificity (Sp), accuracy (Acc), and the area under the curve (AUC), were used (and defined below) for assessment of classification performance. In addition, AUC and Acc refer to the overall system performance, while the remaining indexes are specific to each class, and they measure the generalization ability of the classification algorithm to differentiate events.

Moreover, the Acc includes both test set accuracy and training set accuracy. According to the attribute of the label (positive or negative), the result can generate four basic indexes: true positive (TP), false positive (FP), true negative (TN), and false negative (FN), and in this case, Acc is the radio of the number of correct predicted labels and total number of the labels, thus Acc=(TP+TN)/(TP+TN+FP+FN). Se is the true positive rate and is the probability of incorrectly diagnosing into positive among all positive patients, so Se=TP/(TP+FN). Sp is proportion of incorrectly diagnosing into negative among all negative patients, so Sp=TN/(TN+FP). ROC curve is based on a series of different ways of binary classification (boundary value or decision threshold), with true positive rate (Se) as the ordinate, the false positive rate (1−Sp) as the abscissa, and AUC is defined as the area under the ROC curve, often used to evaluate the classifier with imbalance data. Each fold is tested by a specific classifier with the same parameters as shown in Section 4.1; besides, we also selected the average and standard deviation (SD) of the experimental results to be evaluated, and the results are summarized in Table 4.

From Table 4, a mean Acc of 81.07% from 5-fold cross validation is achieved for the test data. The corresponding Se, Sp, and AUC results are 74.96%, 86.41%, and 0.88. It is worth to note that the results from the fourth fold are low. This is because there is an extremely poor signal quality ECG recording in the fourth fold divided as shown in Table 1, which has significantly different time-frequency features compared to the clean ECG signals. So the results from the folds 1, 2, 3, and 5 are recalculated as shown in Table 4 to exclude the low-quality signal effect. Herein, a mean Acc of 84.85% is achieved for the test data, with the corresponding Se, Sp, and AUC values of 79.05%, 89.99%, and 0.92.

## 5. Discussion and Conclusion

An ECG is widely used in medicine to monitor small electrical changes on the skin of a patient's body arising from the activities of the human heart. Due to the variability and difficulty of AF, traditional detection algorithm cannot be extracted to distinguish obvious characteristics accurately.

In our work, we present a unique architecture of CNN to distinguish AF beats from all other types of ECG beats. MFSWT is adopted to acquire the T-F images of AF and non-AF, respectively, and then, we divide all the data in the MIT-BIH AFDB into training set and test set different from the existing models and build a deep CNN with a total of 12 layers to extract the characters of training set. Finally, the test set is evaluated by the trained model to obtain the performance indexes (including Acc, Se, Sp, and AUC).

Compared with other studies, the difference is that we use all ECG recordings in the MIT-BIH AFDB. However, other studies only selected a part of the recordings for training and testing, and Table 5 shows the comparison between our study and other studies. Obviously, the proposed method does not improve the Acc, Se, and Sp significantly, but this paper uses lots of data to train the model in order to improve the generalization ability of the model. Moreover, we use an important indicator called AUC to evaluate the unbalanced data model and obtained a good evaluation standard.

In short, we proposed a protocol for AF beat detection as follows. (1) Use all the recordings of MIT-BIH Atrial Fibrillation Database for algorithm development and validation. (2) Use 5-fold cross validation to examine the algorithm performance. The results have been registered for Acc, Se, Sp, and AUC. Group the folds by recordings rather than heartbeats to prevent heartbeats of the same patient from appearing in both training and test sets. (3) Use a separate database, for instance AF Database, as an independent test to evaluate the generalization ability of the algorithm. We believe that accurate AF beat recognition can facilitate the detection of AF rhythm. As the first step, AF beat is particularly important. Only by improving the accuracy and generalization of AF beat detection can we more effectively implement AF surveillance.

In addition, more data could be used to evaluate the proposed method; for example, we only focus on one ECG lead, and the study can be extended to two ECG leads. We also can try to use more databases for verification. This algorithm can be used for monitoring and prevention of AF, which has great practical meaning.

## Figures and Tables

**Table 1:** Recordings of grouping conditions.

Fold	Recordings				
1	04015	04126	04936	07879	08405
2	04043	04048	07859	07910	
3	04746	05261	08215	08378	08455
4	04908	06426	07162	08219	08434
5	05091	05121	06453	06995	

**Table 2:** Numbers of the images for testing each fold.

Testing fold	Training		Balanced training		Test	
	Non-AF	AF	Non-AF	AF	Non-AF	AF
1	415,109	294,136	294,136	294,136	123,083	90,403
2	422,935	302,194	302,194	302,194	115,257	82,345
3	424,919	300,509	300,509	300,509	113,273	84,030
4	441,739	314,916	314,916	314,916	96,453	69,623
5	448,066	326,401	326,401	326,401	90,126	58,138

**Table 3:** The optimal CNN specifications designed for the ECG classification problem.

Parameters	Values
Learning rate	0.001
First convolutional layer kernel size	10 *∗* 9
Number of feature maps in the first convolutional and subsampling layer	32
Second convolutional layer kernel size	8 *∗* 7
Number of feature maps in the second convolutional and subsampling layer	20
Third convolutional layer kernel size	9
Number of feature maps in the third convolutional and subsampling layer	16
Subsampling layer kernel size	2
Number of neurons in the first fully connected layer	10
Number of neurons in the second fully connected layer	5
Number of neurons in the third fully connected layer	2
Number of epochs	15
Number of minimal batches	256

**Table 4:** The experimental results.

Fold	Test data				Training data			
	Acc (%)	Se (%)	Sp (%)	AUC	Acc (%)	Se (%)	Sp (%)	AUC
1	86.63	77.95	95.97	0.95	97.59	96.68	98.54	0.99
2	86.82	84.28	88.62	0.93	98.21	97.81	98.62	0.99
3	83.55	78.90	87.38	0.91	97.80	96.77	98.88	0.99
4	**65.93**	**58.59**	**72.08**	**0.70**	**98.26**	**98.51**	**98.00**	**0.98**
5	82.41	75.06	87.99	0.90	98.40	98.56	98.24	0.99
Mean	81.07	74.96	86.41	0.88	98.05	97.67	98.46	0.99
SD	8.68	9.74	8.73	0.10	0.34	0.91	0.34	0
Mean^#^	84.85	79.05	89.99	0.92	98.00	97.46	98.57	0.99
SD^#^	1.92	3.34	3.48	0.02	0.32	0.78	0.23	0

^#^The results only from the average of the folds 1, 2, 3, and 5.

**Table 5:** Comparison with reference studies.

Algorithm	Data	Acc (%)	Se (%)	Sp (%)	AUC
Chen et al. [11]	50 signals	—	89.68	86.16	—
Maji et al. [12]	178 cycles	—	90.48	92.89	—
Ladavich and Ghoraani [13]	14,600 beats	—	90.67	88.87	—
Proposed method	All recordings	81.07	74.96	86.41	0.88
Proposed method	All recordings but excluding one fold with extreme noisy recording	84.85	79.05	89.99	0.92

## Data Availability

The data used to support the findings of this study are available from the open-access MIT-BIH Atrial Fibrillation Database.
